# Wnt3a upregulates brain-derived insulin by increasing NeuroD1 via Wnt/β-catenin signaling in the hypothalamus

**DOI:** 10.1186/s13041-016-0207-5

**Published:** 2016-03-08

**Authors:** Jaemeun Lee, Kyungchan Kim, Seong-Woon Yu, Eun-Kyoung Kim

**Affiliations:** Department of Brain and Cognitive Sciences, Daegu Gyeongbuk Institute of Science and Technology (DGIST), 333 Techno Jungang-daero, Hyeonpung-myeon, Dalseong-gun, Daegu, 42988 South Korea; Neurometabolomics Research Center, Daegu Gyeongbuk Institute of Science and Technology (DGIST), 333 Techno Jungang-daero, Hyeonpung-myeon, Dalseong-gun, Daegu, 42988 South Korea

**Keywords:** Brain-derived insulin, Hypothalamus, Wnt/β-catenin signaling, NeuroD1

## Abstract

**Background:**

Insulin plays diverse roles in the brain. Although insulin produced by pancreatic β-cells that crosses the blood–brain barrier is a major source of brain insulin, recent studies suggest that insulin is also produced locally within the brain. However, the mechanisms underlying the production of brain-derived insulin (BDI) are not yet known.

**Results:**

Here, we examined the effect of Wnt3a on BDI production in a hypothalamic cell line and hypothalamic tissue. In N39 hypothalamic cells, Wnt3a treatment significantly increased the expression of the *Ins2* gene, which encodes the insulin isoform predominant in the mouse brain, by activating Wnt/β-catenin signaling. The concentration of insulin was higher in culture medium of Wnt3a-treated cells than in that of untreated cells. Interestingly, neurogenic differentiation 1 (NeuroD1), a target of Wnt/β-catenin signaling and one of transcription factors for insulin, was also induced by Wnt3a treatment in a time- and dose-dependent manner. In addition, the treatment of BIO, a GSK3 inhibitor, also increased the expression of *Ins2* and *NeuroD1*. Knockdown of NeuroD1 by lentiviral shRNAs reduced the basal expression of *Ins2* and suppressed Wnt3a-induced *Ins2* expression. To confirm the Wnt3a-induced increase in *Ins2* expression in vivo, Wnt3a was injected into the hypothalamus of mice. Wnt3a increased the expression of *NeuroD1* and *Ins2* in the hypothalamus in a manner similar to that observed in vitro.

**Conclusion:**

Taken together, these results suggest that BDI production is regulated by the Wnt/β-catenin/NeuroD1 pathway in the hypothalamus. Our findings will help to unravel the regulation of BDI production in the hypothalamus.

**Electronic supplementary material:**

The online version of this article (doi:10.1186/s13041-016-0207-5) contains supplementary material, which is available to authorized users.

## Background

Recognition of the importance of insulin action in the brain has grown in many aspects such as energy balance, glucose homeostasis, neuronal survival, synapse formation, and cognition [1–4]. In particular, studies on the relationship between metabolic and neurodegenerative diseases emphasized the role of insulin signaling in the brain [5–10]. However, the source of insulin in the brain has not been fully investigated.

Insulin produced in pancreatic β-cells is a major source of plasma insulin. Insulin produced by the pancreas is supplied to the brain from the blood circulation through the blood–brain barrier [11, 12], since insulin can be transported across this barrier [13]. However, not all insulin in the brain comes from the pancreas, because insulin can also be produced in the brain [14, 15]. Insulin mRNA is detected in various regions of the brain both during development and in adults. Rodents have two non-allelic insulin genes, *Ins1* and *Ins2* [14, 15]. In the pancreas, both genes are expressed, with *Ins2* expressed at a higher level [16]. Interestingly, *Ins1* mRNA is not detected in the brain. Instead, *Ins2* mRNA is found in limbic and olfactory regions, hippocampus, and hypothalamus [6, 17–22]. Nevertheless, it is difficult to detect *Ins2* mRNA in the brain because of its quite low expression level. Using single-cell digital PCR, one research group found that the neurogliaform cells in the cerebral cortex express *Ins2* mRNA [23]. To verify the production of insulin in the brain, brain was stained with antibodies against connecting peptide (c-peptide), byproduct of proinsulin processing. C-peptide–positive signal was found in the central nervous system, and the pattern of c-peptide staining showed correlation with that of *Ins2* expression [24, 25]. However, the mechanisms of local production of insulin in the brain, which is called brain-derived insulin (BDI), and its roles remain unclear.

Wnt signaling is involved in brain development [26–28]. Wnt proteins are a diverse family of secreted glycoproteins that act as ligands for receptor-mediated signaling pathways [29]. The three well-characterized Wnt signaling pathways are the canonical Wnt pathway, the non-canonical planar cell polarity pathway, and the non-canonical Wnt/calcium pathway.

The canonical Wnt signaling regulates the expression of insulin in pancreatic β-cells and the development of the pancreas [30–35]. Without Wnt ligands, β-catenin, which enhances the expression of many genes in the nucleus, is degraded by the β-catenin destruction complex [36]. Glycogen synthase kinase 3 (GSK3), a key component of the β-catenin destruction complex, phosphorylates β-catenin at Ser33, Ser37, and Thr41, leading to its degradation by the proteasome [37]. When Wnt ligands bind to Frizzled (Fz) receptor and its coreceptor, low density lipoprotein receptor–related protein (LRP), the β-catenin destruction complex is disrupted by GSK3 inactivation [38]. Consequently, β-catenin accumulates in the cytosol and translocates into the nucleus to enhance the expression of many target genes [39].

The activity of the canonical Wnt signaling is also observed in the adult mice hypothalamus [40, 41]. Wnt-responsive cells are abundant in the paraventricular and arcuate nuclei of the hypothalamus [42, 43]. In the mouse models of metabolic diseases such as diet-induced obese (DIO) mouse model and leptin-deficient (*ob*/*ob*) mouse model, hypothalamic Wnt signaling is disrupted [44]. GSK3 is increased in the hypothalamus of DIO mice, and a GSK3 inhibitor acutely improves glucose metabolism in these mice [44]. In addition, the phosphorylated (active) form of LRP is down-regulated in *ob*/*ob* mice [45]. Down-regulated Wnt signaling is reinstated by leptin treatment [45]. These reports suggest that Wnt signaling is necessary for glucose homeostasis in the hypothalamus. However, the specific mechanism how Wnt signaling modulates metabolism in the hypothalamus has not yet been revealed.

Insulin signaling plays many roles in the brain [46], however, the mechanisms underlying the expression of insulin in the brain have not been discovered yet. In this study, we examined how Wnt signaling regulates the production of insulin in the hypothalamus. The results of our research will help to reveal the mechanisms underlying the regulation of BDI production in the hypothalamus.

## Results

### Wnt3a increases the levels of *Ins2* mRNA and protein in N39 cells

We used the immortalized mouse hypothalamic neuronal cell line N39 (mHypoE-39) to investigate the mechanism of BDI production, because N39 cells express *Ins2* [14]. Consistent with the previous findings [14], *Ins2* mRNA but not *Ins1* mRNA was detected by quantitative real-time PCR (qRT-PCR) in N39 cells (data not shown). Wnt3a increases insulin expression in pancreatic β-cells and neuronal progenitor cells (derived from adult hippocampus or the olfactory bulb) via Wnt/β-catenin signaling [35, 47]. Therefore, we tested whether Wnt3a can induce insulin production in N39 cells.

**Fig. 1:**
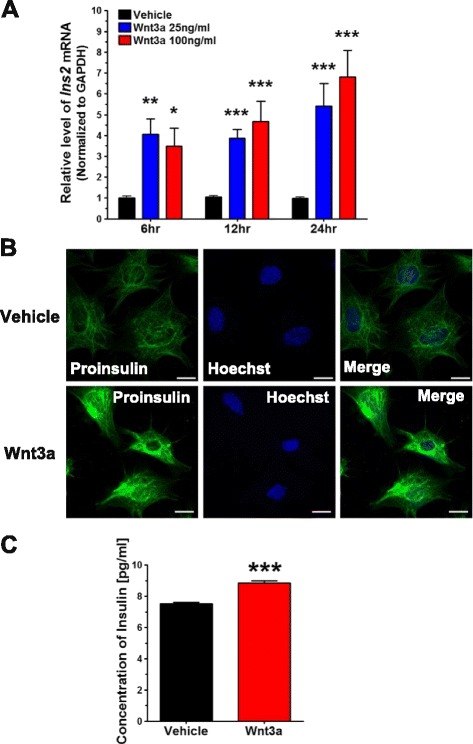
Wnt3a upregulates insulin synthesis and secretion from N39, a hypothalamic neuronal cell line. **a** N39 cells were treated with vehicle (PBS) or Wnt3a (25 or 100 ng/mL) for 6, 12, and 24 h, and the levels of *Ins2* mRNA were measured by qRT-PCR and normalized to the levels of *GAPDH* mRNA (*n* = 23). **b** N39 cells were treated with vehicle or Wnt3a (100 ng/mL) for 24 h, and the induction of insulin was examined by immunofluorescence analysis with an antibody against proinsulin. Nuclei were stained with Hoechst 33342 dye. Scale bar, 20 μm. **c** Culture media were collected for 24 h after treatment with vehicle or Wnt3a (100 ng/mL), concentrated in Vivaspin columns, and insulin concentrations were measured by ELISA (*n* = 6). Data are means + SEM. *p < 0.05, **p < 0.01, ***p < 0.001 compared with vehicle control at each time point

N39 cells were treated with Wnt3a (25 or 100 ng/mL) for 6, 12, or 24 h. After Wnt3a treatment for 6 h, the *Ins2* mRNA level increased significantly (by 4.1-fold at 25 ng/mL and 3.5-fold at 100 ng/mL Wnt3a; Fig. 1a), although this increase was not dose-dependent. After 12 h, Wnt3a increased the *Ins2* mRNA level by 3.9-fold at 25 ng/mL and 4.7-fold at 100 ng/mL. After 24 h, the *Ins2* mRNA level was increased by 5.4-fold at 25 ng/mL and 6.8-fold at 100 ng/mL of Wnt3a compared to vehicle-treated cells. Thus, the *Ins2* mRNA levels were significantly increased in Wnt3a-treated N39 cells in a time-dependent manner at 12 and 24 h.

To confirm the expression of insulin, we checked its protein level using immunofluorescence analysis with an antibody against proinsulin and found stronger proinsulin-positive signals in N39 cells treated with Wnt3a (100 ng/mL) for 24 h than in vehicle-treated cells (Fig. 1b).

To further verify the production of insulin in Wnt3a-treated N39 cells, culture medium was collected after Wnt3a treatment for 24 h, concentrated, and the concentration of immuno-reactive insulin was measured with ELISA. The concentration of insulin in culture medium was significantly increased (by 17.4 %) in cells treated with Wnt3a (100 ng/mL) as compared to vehicle-treated cells (Fig. 1c).

Taken together, our results indicate that Wnt3a increases the levels of insulin mRNA and protein in N39 cells. Furthermore, Wnt3a also increases secretion of insulin from N39 cells.

### Wnt3a induces insulin production in N39 cells through the canonical Wnt/β-catenin signaling

In the canonical Wnt signaling, Wnt ligands bind to Fz receptor and LRP, and this binding results in the stabilization, accumulation, and nuclear translocation of β-catenin [48, 49]. To determine whether Wnt3a induced *Ins2* through the canonical Wnt signaling, we measured the level of β-catenin in Wnt3a-treated N39 cells.

**Fig. 2:**
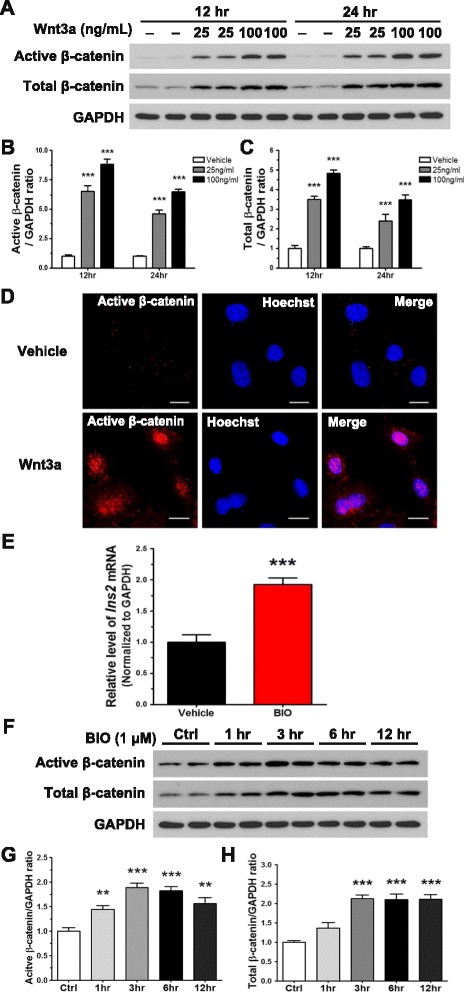
Wnt3a induces the expression of *Ins2* by activating Wnt/β-catenin signaling. **a** N39 cells were treated with vehicle or Wnt3a (25 or 100 ng/mL) for 12 and 24 h. Active (Non-phospho) β-catenin and total β-catenin were detected using immunoblot assay. **b** and **c** The intensity of bands shown in (**a**) was quantified by using the ImageJ software with normalization to GAPDH (*n* = 6). **d** N39 cells were treated with vehicle or Wnt3a (100 ng/mL) for 24 h, and active β-catenin was examined by immunofluorescence analysis; Hoechst 33342 dye was used for nuclear staining. Scale bar, 20 μm. **e** N39 cells were treated with 1 μM BIO, a GSK3 inhibitor, for 12 h, and the level of *Ins2* mRNA was measured by qRT-PCR (*n* = 9). **f** N39 cells were treated with vehicle (DMSO) or 1 μM BIO for 1, 3, 6, and 12 h to observe accumulation of active β-catenin and total β-catenin (*n* = 6). **g** and **h** Quantification of immunoblot data in (**f**) using the ImageJ software was performed with normalization to GAPDH. Data are means + SEM. **p < 0.01, ***p < 0.001 compared with vehicle treatment

Immunoblot analysis (Fig. 2a-c) showed that Wnt3a strongly stabilized β-catenin in a dose-dependent manner, as shown by a significant increase in the levels of active β-catenin, in which the Ser33, Ser37, and Thr41 residues are dephosphorylated. Consequently, total β-catenin also accumulated in a dose-dependent manner. Consistent with the immunoblotting data, immunofluorescence analysis showed that Wnt3a treatment resulted in the accumulation of active β-catenin (Fig. 2d). Furthermore, active β-catenin was clearly co-localized with Hoechst 33342, a nuclear marker, in Wnt3a-treated N39 cells.

To ascertain the role of GSK3 in insulin production stimulated by Wnt3a treatment in N39 cells, we treated them with 6-bromoindirubin-3′-oxime (BIO), a specific GSK3 inhibitor. BIO inhibits GSK3 by interacting with its ATP binding pocket, leading to activation of the canonical Wnt signaling in human and mouse embryonic stem cells [50, 51]. BIO treatment increased the level of *Ins2* mRNA by 2-fold after treatment for 12 h (Fig. 2e). As expected, the levels of both active and total β-catenin increased by up to 2-fold after BIO treatment for 3 h, and the increased level was maintained after BIO treatment for 6 and 12 h (Fig. 2f-h). The induction of *Ins2* and accumulation of β-catenin upon BIO treatment showed trends similar to those of the effects of Wnt3a treatment. Collectively, these data indicate that Wnt3a increases insulin production by activating β-catenin and it might be mediated by inactivation of GSK3.

### Induction of insulin production by Wnt3a depends on NeuroD1

To unravel the mechanism of insulin production in N39 cells, we investigated the downstream targets of Wnt/β-catenin signaling. Among many targets of this pathway, we tested NeuroD1, which is known as a candidate transcription factor involved in the expression of the insulin genes in the pancreas [52].

**Fig. 3:**
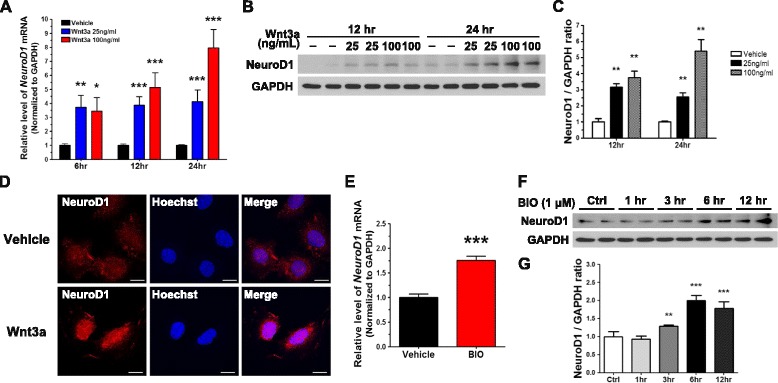
Wnt3a increases the expression of NeuroD1 by activating Wnt/β-catenin signaling in N39 cells. **a** *NeuroD1* mRNA levels were quantified using qRT-PCR after vehicle or Wnt3a (25 or 100 ng/mL) treatment for 6, 12, and 24 h (*n* = 23). **b** NeuroD1 protein levels were measured by immunoblot assay after vehicle or Wnt3a (20 or 100 ng/mL) treatment for 12 and 24 h. **c** The intensity of bands shown in (**b**) was quantified by using ImageJ with normalization to GAPDH (*n* = 6). **d** After treatment with vehicle or Wnt3a (100 ng/mL) for 24 h, NeuroD1 was examined using the immunofluorescence assay; the nuclei were stained with Hoechst 33342 dye. Scale bar, 20 μm. **e** The level of *NeuroD1* mRNA was determined by qRT-PCR 12 h after treatment with 1 μM BIO (*n* = 9). **f** Cells were treated with 1 μM BIO, and the level of NeuroD1 was measured by immunoblot assay after treatment for 1, 3, 6, and 12 h. **g** The intensity of NeuroD1 bands in (**f**) was quantified using the ImageJ software and normalized to GAPDH (*n* = 6). Data are means + SEM. *p < 0.05, **p < 0.01, ***p < 0.001 compared with vehicle treatment

We measured the *NeuroD1* mRNA level at 6, 12, and 24 h in N39 cells treated with Wnt3a (25 or 100 ng/mL). At 6 h, the level of *NeuroD1* mRNA was significantly increased in comparison with vehicle-treated N39 (by 3.7-fold at 25 ng/mL and 3.5-fold at 100 ng/mL Wnt3a; Fig. 3a). At 12 h, *NeuroD1* mRNA was upregulated by 3.9-fold at 25 ng/mL and 5.1-fold at 100 ng/mL Wnt3a. At 24 h, the effects on *NeuroD1* mRNA were even more profound (4.1-fold increase at 25 ng/mL and 8.0-fold increase at 100 ng/mL Wnt3a). The induction of *NeuroD1* mRNA by Wnt3a showed a pattern similar to that of *Ins2* mRNA: after Wnt3a treatment for 6 h, *Ins2* and *NeuroD1* did not show dose-dependent effects, whereas both the *Ins2* and *NeuroD1* mRNA levels were significantly increased in a dose-dependent manner at 12 and 24 h. Interestingly, at 1.5 h after Wnt3a (100 ng/mL) treatment, the mRNA level of *NeuroD1* was increased significantly whereas that of *Ins2* was not increased yet (Additional file 1: Figure S1A and S1B). To validate the effect of Wnt3a on NeuroD1 expression, we determined the level of the NeuroD1 protein in Wnt3a-treated N39 cells by immunoblotting (Fig. 3b and c). At 12 h, the NeuroD1 level was significantly increased (by 3.2 fold at 25 ng/mL and 3.8 fold at 100 ng/mL Wnt3a). At 24 h, the increase was dose-dependent (2.6 fold at 25 ng/mL and 5.4 fold at 100 ng/mL Wnt3a).

NeuroD1 was detectable in N39 cells by immunofluorescence analysis (Fig. 3d). When the cells were treated with Wnt3a (100 ng/mL) for 24 h, the intensity of the NeuroD1-positive signal was apparently greater. Co-localized with Hoechst 33342 demonstrated Wnt3a-induced nuclear localization of NeuroD1. These data demonstrate that Wnt3a increases both the expression of NeuroD1 and its translocation into the nucleus in N39 cells.

We also treated N39 cells with BIO to determine whether the induction of NeuroD1 is mediated by GSK3-dependent Wnt/β-catenin signaling. The level of *NeuroD1* mRNA was increased by 2-fold in cells treated with BIO for 12 h (Fig. 3e). In a similar manner, BIO treatment for 3, 6, or 12 h significantly increased the levels of the NeuroD1 protein (by up to 2-fold compared to vehicle-treated N39 cells; Fig. 3f and g). These results suggest that Wnt/β-catenin signaling increases the expression of NeuroD1 through GSK3 inhibition.

**Fig. 4:**
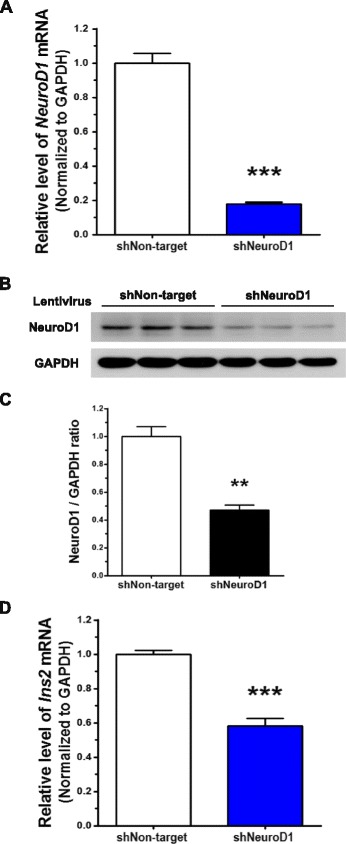
Knockdown of NeuroD1 reduces the expression of *Ins2*. NeuroD1 was knocked down by infecting N39 cells with lentivirus containing shRNA (shNeuroD1) for 72 h. **a** Knockdown efficiency was determined by examining the levels of *NeuroD1* mRNA using qRT-PCR (*n* = 23). **b** The level of NeuroD1 protein was assessed by immunoblot assay, and **c** the intensity of NeuroD1 bands was quantified using the ImageJ software with normalization to GAPDH (*n* = 6). **d** The basal expression of *Ins2* was determined by qRT-PCR after infection with shRNA lentivirus against NeuroD1 for 72 h (*n* = 12). Data are means + SEM. **p < 0.01, ***p < 0.001 compared with non-targeting shRNA lentivirus-infected cells (shNon-target)

To determine whether the induction of BDI by Wnt3a is NeuroD1-dependent, knockdown experiments were designed using a lentiviral vector system. Among 5 candidate short-hairpin RNA (shRNA) constructs designed to target NeuroD1, two shRNAs were chosen because of their high knockdown efficiency and used in combination for NeuroD1 knockdown (data not shown). Lentiviruses expressing NeuroD1 shRNAs significantly suppressed the expression of NeuroD1: by 80 % at the mRNA level (Fig. 4a) and by 50 % at the protein level after 72 h infection (Fig. 4b and c) compared to lentivirus expressing non-targeting shRNA. Interestingly, NeuroD1 knockdown reduced the expression of *Ins2* by 40 % of that in non-targeting shRNA lentivirus–infected cells in the absence of Wnt3a, indicating that NeuroD1 is involved in maintaining the expression of *Ins2* in the basal state (Fig. 4d).

**Fig. 5:**
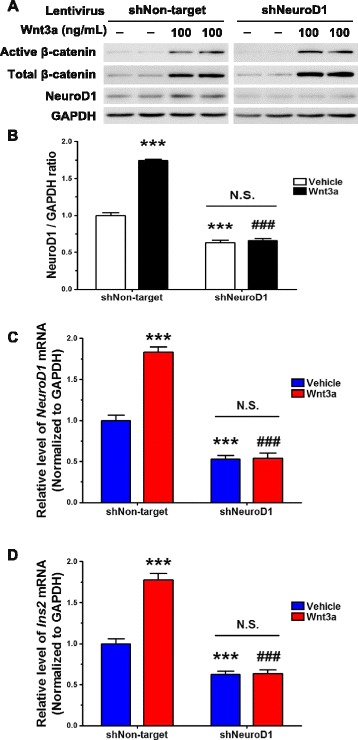
Wnt3a-induced *Ins2* upregulation is blocked by knockdown of NeuroD1. NeuroD1 was knocked down by infecting N39 cells with lentivirus containing shRNA for 72 h. **a** N39 cells were infected by lentiviral shRNA against NeuroD1 for 48 h and then treated with Wnt3a (100 ng/mL) for 24 h. The levels of active and total β-catenin and NeuroD1 protein were determined by immunoblot assay. **b** The intensity of NeuroD1 signal in (**a**) was quantified using the ImageJ software (*n* = 4). The levels of *NeuroD1* mRNA (**c**) and *Ins2* mRNA (**d**) were measured by qRT-PCR in N39 cells that were infected with lentiviral non-targeting or NeuroD1 shRNA for 48 h and then treated with Wnt3a (100 ng/mL) for 24 h (*n* = 9). Data are means + SEM. ***p < 0.001 compared with vehicle treatment in shNon-target. ###*p* < 0.001 compared with Wnt3a treatment in shNon-target. N.S., not significant within shNeuroD1 samples

In addition, we treated N39 cells, in which NeuroD1 was knocked down, with Wnt3a (100 ng/mL) to examine any changes in the induction of *Ins2*. As shown in Fig. 5a and b, Wnt3a treatment resulted in the accumulation of active and total β-catenin in cells infected with lentiviruses expressing either non-targeting shRNA or NeuroD1 shRNA. However, as demonstrated by using qRT-PCR, the NeuroD1 transcript was not upregulated by Wnt3a in N39 cells with knocked down NeuroD1, although it was significantly increased in non-targeting shRNA lentivirus–infected N39 cells (Fig. 5c). Furthermore, Wnt3a treatment failed to induce *Ins2* in N39 cells with knocked down NeuroD1 whereas Wnt3a significantly increased the level of *Ins2* mRNA in non-targeting shRNA lentivirus–infected N39 cells (Fig. 5d).

Taken together, these results suggest that NeuroD1 is a key transcription factor for Wnt3a-induced *Ins2* expression through Wnt/β-catenin signaling in N39 cells.

### Wnt3a administration induces the expression of *Ins2* and *NeuroD1* in the hypothalamus

**Fig. 6:**
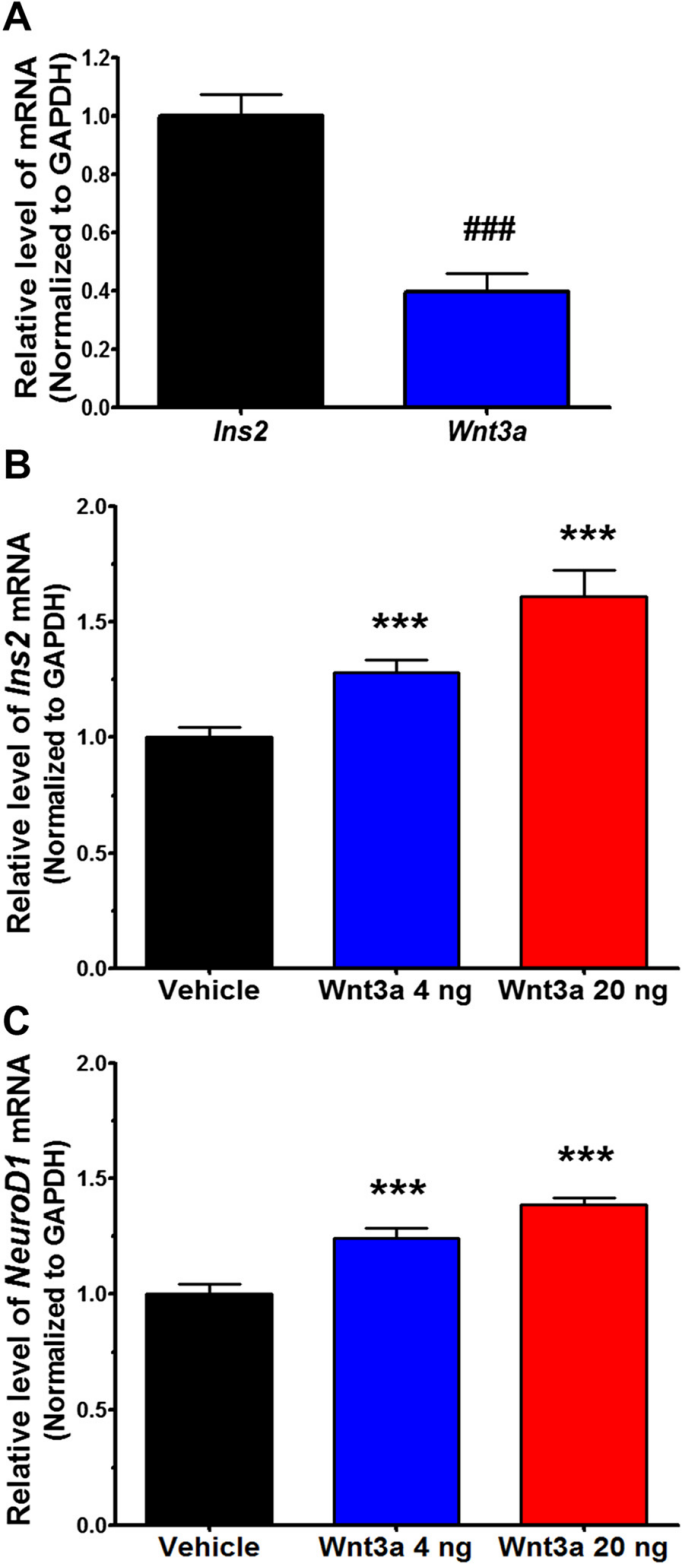
Wnt3a upregulates *NeuroD1* and *Ins2* in the mouse hypothalamus. The basal mRNA levels of *Ins2* and *Wnt3a* were measured by qRT-PCR in hypothalamic tissues from C57BL/6 male mice (*n* = 6−9) (**a**). C57BL/6 male mice received icv injections of vehicle or Wnt3a (4 or 20 ng), and hypothalamic tissues were dissected after 24 h. The mRNA levels of *Ins2* (**b**) and *NeuroD1* (**c**) were measured by qRT-PCR (*n* = 6−9). Data are means + SEM. ###*p* < 0.001 compared with *Ins2* mRNA, ***p < 0.001 compared with vehicle administration

Many reports showed Wnt activity in the adult hypothalamus [40–43]. Autocrine and/or paracrine Wnt ligands may be responsible for Wnt activity in the hypothalamus. To test Wnt3a-induced BDI production in the hypothalamus in vivo, we first measured the mRNA level of *Wnt3a* by qRT-PCR in the hypothalamic tissues (Fig. 6a). Although the amount of *Wnt3a* mRNA was relatively lower than that of *Ins2*, the expression of *Wnt3a* was detected in the hypothalamus.

To examine the effect of Wnt3a treatment on the synthesis of *Ins2* mRNA in hypothalamic tissue, we introduced Wnt3a into the hypothalamus by intracerebroventricular (icv) injection and collected hypothalamic tissues for analysis 24 h after injection. Administration of Wnt3a (4 or 20 ng) significantly increased the levels of *Ins2* mRNA (by 1.3-fold and 1.6-fold, respectively; Fig. 6b). Interestingly, the levels of *NeuroD1* mRNA were also upregulated (Fig. 6c). These results indicate that Wnt3a increases the expression of *Ins2* and *NeuroD1* in the hypothalamus.

## Discussion

**Fig. 7:**
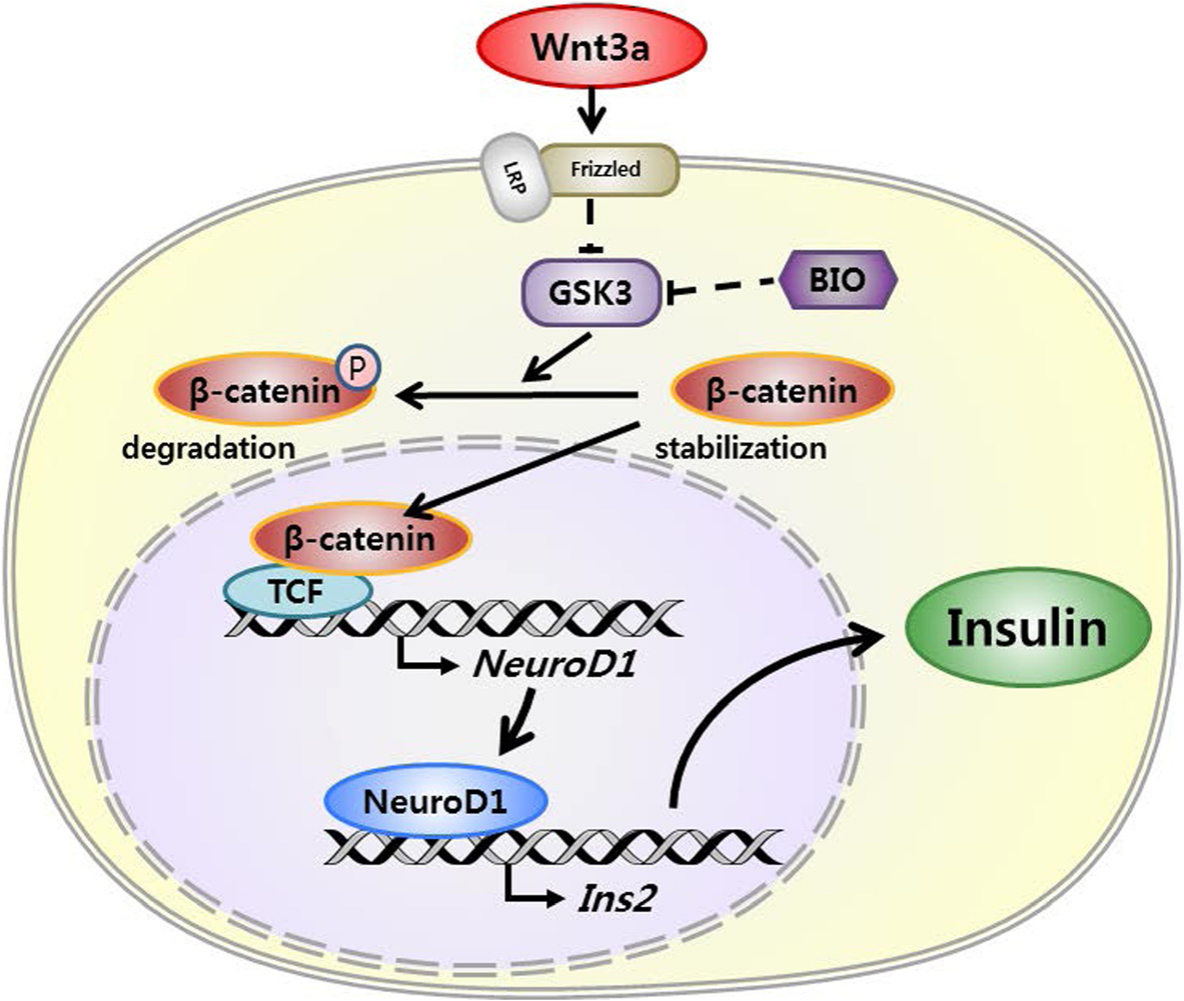
The proposed regulatory mechanism of insulin expression by the Wnt/β-catenin/NeuroD1 pathway in the hypothalamus. Wnt3a might bind to LRP and Frizzed receptor to activate the canonical Wnt pathway. GSK3 phosphorylates β-catenin and induces its degradation; Wnt3a inhibits GSK3, leading to β-catenin accumulation. Accumulated β-catenin translocates into the nucleus and enhances the expression of NeuroD1 with TCF. Up-regulated NeuroD1 also translocates into the nucleus, where it induces the production of insulin

Despite accumulating evidence about BDI, the specific mechanism of BDI production is not yet well understood. In particular, hypothalamic insulin production has not been reported. Considering that the hypothalamus has a large number of neurosecretory cells, which produce and secrete diverse neuropeptides involved in maintaining homeostasis, it is plausible that BDI plays a specific role in maintaining energy homeostasis and regulating metabolism in the hypothalamus. In this study, we demonstrated that Wnt3a increases insulin production in the hypothalamus by activating Wnt/β-catenin signaling and NeuroD1 induction. Inhibition of GSK3, which phosphorylates β-catenin and thus promotes its degradation, leads to accumulation of β-catenin in the cytosol. Accumulated β-catenin translocates into the nucleus, where it enhances the expression of NeuroD1 with T-cell factor (TCF). Synthesized NeuroD1 also moves into the nucleus and induces insulin production (Fig. 7).

Hippocampus and olfactory regions are known as brain regions that express insulin higher than other brain regions [15, 35]. The *Ins2* mRNA level is lower in the hypothalamus than in the hippocampus [14, 17]. However, insulin signaling plays important roles in controlling food intake, glucose metabolism, and energy expenditure in the hypothalamus [53, 54]. Nevertheless the expression level is lower than other regions, we hypothesized that BDI synthesized in the hypothalamus could regulate metabolism by acting on the hypothalamic neurocircuits.

The activation of Wnt/β-catenin signaling enhances the transcription of downstream genes via the formation of a complex between active β-catenin and TCF [55]. Our results demonstrate not only the accumulation of active and total β-catenin (Fig. 2a-c), but also the translocation of active β-catenin into the nucleus induced by Wnt3a in N39 cells (Fig. 2d). Although we did not examine the activity of the TCF complex, these results indicate activation of Wnt/β-catenin signaling in these cells. The level of NeuroD1, a downstream target of Wnt/β-catenin signaling, was increased in Wnt3a-treated N39 cells (Fig. 3a-d). This increase in NeuroD1 expression supports that Wnt3a-induced accumulation of active β-catenin enhances the transcription of NeuroD1 gene.

To determine whether GSK3 is involved in Wnt3a-induced *Ins2* upregulation, we treated BIO to inhibit GSK3 (Fig. 2e-h, Fig. 3e-g). BIO treatment resulted in accumulation of β-catenin, and increases in the expression of *NeuroD1* and *Ins2*, suggesting that GSK3 plays a role in the upregulation of *Ins2* by Wnt/β-catenin signaling. The phosphorylation of GSK3 at Ser9 residue is one of mechanisms for GSK3 inactivation [56, 57]. However, we could not observe any changes in the level of phospho-GSK3 (Ser9) when N39 was treated with Wnt3a (data not shown). Therefore, it is likely that GSK3 inhibition by Wnt3a is mediated by not phosphorylation of Ser9 but other mechanisms in our study.

NeuroD1 is a basic helix-loop-helix transcription factor that plays a role in facilitating differentiation of neurons [58, 59], and its gene is a target of Wnt/β-catenin signaling in the brain [52, 60]. NeuroD1 also binds to the insulin promoter to increase the expression [61, 62]. Although an increase in *Ins2* mRNA levels in Wnt3a-injected hypothalamic tissues was smaller than that in N39 cells, the levels of both *NeuroD1* and *Ins2* mRNAs increased in the hypothalamus of Wnt3a-injected mice. Taken together, it is likely that insulin production induced by Wnt3a, which is well known to occur in the pancreas, is conserved in the hypothalamus.

The mechanism of insulin secretion in neurons is not yet revealed. Mechanistic studies on insulin secretion in vivo are challenging, because it is difficult to pinpoint the origin of insulin in the brain; the confounding factor is that insulin produced in the pancreas is delivered by blood circulation to the brain in vivo. Furthermore, the basal expression of insulin is not high enough to study the mechanism of its secretion in in vitro systems. Insulin-overexpressing N39 cells as an in vitro model might be useful to investigate the mechanism of insulin secretion in the future studies.

The neuron-specific insulin receptor knockout (NIRKO) mice resulted in increased food intake and moderate diet-induced obesity, demonstrating the anti-obesity role of insulin [2]. However, insulin receptor (IR) knockout mice specifically deleted in agouti-related peptide (AgRP)- or pro-opiomelanocortin (POMC)-expressing neuron did not show a significant difference in energy homeostasis, especially food intake [63]. Moreover, deletion of IR in steroidogenic factor 1 (SF1)-expressing neurons of the ventromedial hypothalamus had no effect on body weight and food intake [64]. The different phenotypes among mice models suggest that the roles of insulin through IR in the brain are diverse in a cell-type specific manner. Therefore, it might be important to identify the subpopulation of hypothalamic neurons that specifically express BDI in order to investigate the role of BDI in the future study. It would be interesting to investigate whether Wnt3a-induced insulin production from the specific neurons in the hypothalamus can regulate the food intake and protect from obesity.

## Conclusions

We demonstrate that BDI is regulated by Wnt/β-catenin signaling in the hypothalamus. It is possible that insulin production in the hypothalamus or other brain areas changes in metabolic diseases. Further studies focused on the regulation of BDI production in the disease models with disrupted hypothalamic Wnt/β-catenin signaling will be needed to elucidate the pathophysiological roles of BDI.

## Methods

### Cell culture and treatments

Immortalized mouse hypothalamic cell line N39 (mHypoE-39) was obtained from CELLutions Biosystems and maintained in Dulbecco’s modified Eagle medium (DMEM) (Sigma) supplemented with 10 % fetal bovine serum (FBS) (Hyclone Laboratories) and 1 % penicillin/streptomycin (GIBCO). Lenti-X™ 293 T cell line was purchased from Clontech and cultured in DMEM supplemented with 10 % FBS and 1 % penicillin/streptomycin. Recombinant mouse Wnt3a (1324-WN, R&D Systems) was dissolved in phosphate-buffered saline (PBS) containing 0.2 % BSA. BIO (Sigma), a specific GSK3 inhibitor, was dissolved in dimethyl sulfoxide (DMSO) at 1 mM concentration.

### Animals

Animal studies were performed in accordance with the guidelines on care and use as approved by the DGIST Institutional Animal Care and Use Committee. C57BL/6 mice were obtained from Koatech; 8-week-old males were used for experiments. Mice were housed in groups of 3–5 under a 12/12 h light/dark cycle (lights on from 6:00 to 18:00) in an individually ventilated cage Innorack (Innovive).

### Administration of Wnt3a and preparation of brain tissues

Wnt3a was introduced into the hypothalamus by icv injection of 2 μL of PBS containing Wnt3a (4 or 20 ng) into the third ventricles. Mice were euthanized with CO_2_ gas supplied for 3–5 min 24 h after Wnt3a administration. Brain was quickly removed, placed on ice, and the hypothalamus was dissected using as landmarks the optic chiasm and the mammillary bodies to depth of ~ 2 mm [65]. Hypothalamic tissues were frozen in liquid nitrogen and ground mechanically on dry ice.

### RNA extraction and gene expression analysis by quantitative real-time PCR

Total RNA was isolated using TRIzol (Invitrogen) and chloroform (Sigma) from N39 cells and ground brain tissues. For qRT-PCR analysis, 3 μg of total RNA from each sample was reverse-transcribed using a Reverse Transcription System (Promega). Synthesized cDNA was diluted 1:5 with water, and 2 μl was used as a template for qRT-PCR. Primers were designed as follows: *Ins1* Forward, 5′-AGAGACCATCAGCAAGCAGGTCA-3′; *Ins1* Reverse, 5′-TACCAGGTGGGGACCACAAAGA-3′; *Ins2* Forward, 5′-GTGACCTTCAGACCTTGGCACTG-3′; *Ins2* Reverse, 5′-AGGCTGGGTAGTGGTGGGTCTAG-3′; *NeuroD1* Forward, 5′-TGACCTTTCCCATGCTGAAT-3′; *NeuroD1* Reverse, 5′-AAGTGCTAAGGCAACGCAAT-3′; *GAPDH* Forward, 5′-GTCAATGAAGGGGTCGTTGATGG-3′; and *GAPDH* Reverse, 5′-TCGTCCCGTAGACAAAATGGTGA-3′. qRT-PCR was performed according to the SYBR Green protocol (SYBR Premix Ex Taq, TaKaRa) in a Bio-Rad CFX-96 machine. Annealing and extension were done at 63 °C.

### Enzyme-linked immunosorbent assay

Culture medium from Wnt3a-treated N39 cells was collected and filtered with a 0.22 μm syringe filter. Vivaspin 2 (2000 MWCO Hydrosart; Sartorius) was used to concentrate 3 mL of medium to 35 μL. Insulin concentrations in concentrated media were measured by using an ELISA kit, which detects only mature insulin, purchased from ALPCO Diagnostics.

### Immunoblot analysis

N39 cells were plated at a density of 1.0 × 10^5^/mL in 6-well plates. After appropriate treatments, cells were washed with ice-cold PBS and harvested in lysis buffer (50 mM Tris-HCl, pH 7.4, 250 mM sucrose, 5 mM sodium pyrophosphate, 1 mM EDTA, 1 mM EGTA, 1 % Triton X-100, 0.1 mM benzamidine, 10 μg/mL leupeptin, 1 mM DTT, 0.5 mM PMSF, 50 mM NaF, 1X protease inhibitor cocktail (Calbiochem), 1X phosphatase inhibitor cocktail (Sigma). Protein was extracted from hypothalamic tissues with the same lysis buffer. Lysates (20 μg total protein) were separated on 10 % SDS-polyacrylamide gels and blotted onto polyvinylidene difluoride membranes for 40 min at 20 V in transfer buffer containing 25 mM Tris base and 192 mM glycine. The membranes were blocked with 5 % skim milk for 3 h and then incubated with primary antibodies against non-phospho β-catenin (1:3000; Cell Signaling), total β-catenin (1:3000; Cell Signaling), NeuroD1 (1:3000; AbCam), or glyceraldehyde-3-phosphate dehydrogenase (GAPDH) (1:5000; Cell Signaling) at 4 °C overnight. After three washes with Tris-buffered saline with 0.1 % of Tween 20 (TBST), the membranes were incubated with horseradish peroxidase–linked secondary antibody and washed with TBST three times and visualized by SuperSignal West Pico Chemiluminescent Substrate (Thermo) according to the manufacturer’s procedure. The intensity of bands was quantified using NIH ImageJ analysis software.

### Immunofluorescence analysis

N39 cells were seeded on poly-l-lysine–coated coverslips. After treatment with Wnt3a for 24 h, cells were washed with PBS, fixed with 4 % paraformaldehyde and permeabilized in 0.2 % Triton X-100, 0.1 M glycine. The cells were then washed with PBS, blocked with BSA containing antibody diluent (Invitrogen) at room temperature (RT) and incubated with antibodies against proinsulin (1:50; R&D Systems), non-phospho β-catenin (1:500; Cell Signaling), and NeuroD1 (1:200; Santa Cruz) at 4 °C. Coverslips were incubated with secondary antibodies for 2 h at RT, and Hoechst 33342 (Invitrogen) was used for nuclear staining for 10 min at RT. ProLong Diamond Antifade Mountant (Invitrogen) was used for mounting coverslips. Each slide was analyzed with an LSM700 confocal microscope (Carl Zeiss). Images were analyzed with ZEN2012 software (Carl Zeiss).

### Preperation of lentiviral shRNAs

Five TRC Lentiviral Non-targeting shRNAs control (#RHS6848) and mouse NeuroD1 shRNAs (81773-81777, Dharmacon) were cloned into the pLKO.1 lentiviral vector. The pMD2.G and psPAX2 vectors were used as an envelope and packaging vector, respectively, to produce lentivirus in the Lenti-X 293 T cell line (Clontech). Transient transfections were performed with TurboFect Transfection Reagent (Thermo) according to the manufacturer’s protocol. Cell supernatants containing virus were collected 72 h post-transfection and filtered through 0.45 μm syringe filters. Lentiviral vectors were concentrated by ultracentrifugation at 40,000 × *g* for 90 min at 4 °C. Viral pellets were suspended in PBS.

### Statistical analysis

Data were analyzed with GraphPad Prism software (GraphPad Software). Statistical analysis was performed using unpaired *t*-test.

## Additional file

Additional file 1:
**Supplemental Figure S1. Wnt3a induces** ***NeoruD1*** **prior to upregulation of** ***Ins2*** **.** N39 cells were treated with vehicle (PBS) or Wnt3a (25 or 100 ng/mL) for 1.5, 3 and 4.5h, and the levels of *NeoruD1* mRNA (A) and *Ins2* mRNA (B) were measured by qRT-PCR and normalized to the levels of GAPDH mRNA (n=9). Data are means + SEM. **p* < 0.05, ** *p* < 0.01, *** *p* < 0.001 compared with vehicle control at each time point. (JPG 976 kb)

## Abbreviations

AgRP: agouti-related peptide
BDI: brain-derived insulin
DIO: diet-induced obese
Fz: frizzled
GSK3: glycogen synthase kinase 3
IR: insulin receptor
LRP: low density lipoprotein receptor-related protein
N39 cell: mouse hypothalamic neuronal cell
NeuroD1: neurogenic differentiation 1
NIRKO: neuron-specific insulin receptor knockout
*ob/ob*: leptin-deficient
POMC: pro-opiomelanocortin
SF1: steroidogenic factor 1
TCF: T-cell factor
