# Supplementary figures and images for: Wnt3a upregulates brain-derived insulin by increasing NeuroD1 via Wnt/β-catenin signaling in the hypothalamus
Source: Mol Brain. 2016 Mar 8;9:24. doi: 10.1186/s13041-016-0207-5 (PMC4782570; doi:10.1186/s13041-016-0207-5)

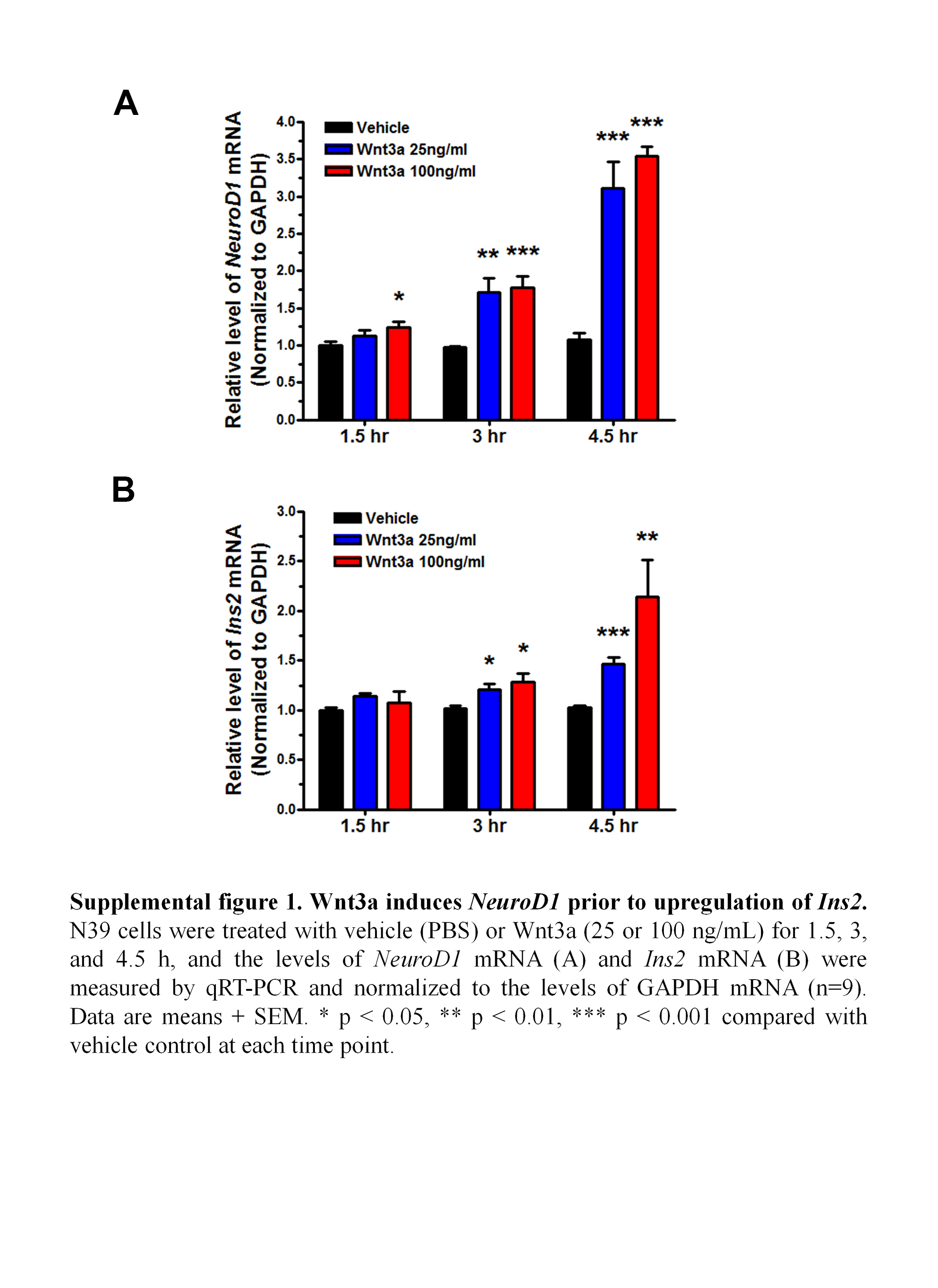

Supplement: Additional file 1: — Supplemental Figure S1. Wnt3a induces NeoruD1 prior to upregulation of Ins2 . N39 cells were treated with vehicle (PBS) or Wnt3a (25 or 100 ng/mL) for 1.5, 3 and 4.5h, and the levels of NeoruD1 mRNA (A) and Ins2 mRNA (B) were measured by qRT-PCR and normalized to the levels of GAPDH mRNA (n=9). Data are means + SEM. *p < 0.05, ** p < 0.01, *** p < 0.001 compared with vehicle control at each time point. (JPG 976 kb) [file 13041_2016_207_MOESM1_ESM.jpg]
